# Volume Thirteen

**Published:** 1896-03

**Authors:** 


### EDITORIAL.

The office of the Business Manager of THE JOURNAL is Nos. 311 and 312 Fitten building.

The editors are not responsible for views expressed by contributors.

Articles for publication should reach this office not later than the 15th instant.

Address all communications, and make all remittances payable to THE ATLANTA MEDICAL AND SURGICAL JOURNAL, Atlanta, Ga.

Reprints of articles will be furnished, when desired, at a nominal cost. Requests for the same should always be made *on the manuscript.*

### VOLUME THIRTEEN.

This issue is the beginning of our thirteenth volume under the new series. We need not at this time say more to our readers than to repeat the assurances made in the past, that we intend to give them the best journal to be had at the popular price of two dollars per annum. As we have lately made some editorial remarks upon journalism in general, we may now say of this JOURNAL in particular, that it is an independent and fearless medical publication, owned and conducted by regular physicians, and is not the organ or adjunct of any publishing house, medical college, or medical society. No medical journal can afford to be hampered by any such affiliations if it wishes to enjoy the privileges of free and independent action and opinion and to accomplish the full purposes of journalism.

We beg to thank our good friends who have encouraged and assisted us in our management of this JOURNAL in the past, and to request a continuance of their kind offices in the future.

The JOURNAL is in a prosperous condition; our subscription and advertising business increases every year, and we intend to keep it steadily on the up-grade.

